# Comprehensive molecular characterization and comparison of venom proteins and transcripts in three *Gloydius* species from South Korea

**DOI:** 10.1038/s41598-026-40454-1

**Published:** 2026-03-06

**Authors:** Hyo Sun Park, Jeong Mi Moon, Byeong Jo Chun, Yerin Min, Yong Soo Cho, Kwang-Hyun Park, Sujin Lee, Yeonjong Koo

**Affiliations:** 1 Department of Agricultural Biological Chemistry, Chonnam National University, Gwangju, 61186 Republic of Korea; 2 Department of Emergency Medicine, Chonnam National University Medical School, Gwangju, 61469 Republic of Korea; 3 Department of Emergency Medical Rescue, Nambu University, Gwangju, 62271 Republic of Korea; 4 School of Earth Sciences and Environmental Engineering, Gwangju Institute of Science and Technology (GIST), Gwangju, 61005 Republic of Korea; 5 Department of Biomaterials Convergence, Chonnam National University, Gwangju, 61186 Republic of Korea

**Keywords:** Viper, Gloydius, Snake venom, Proteomics, Transcriptomics, SVMP, Biochemistry, Biological techniques, Biotechnology

## Abstract

In Korea, *Gloydius brevicaudus*, *G. ussuriensis*, and *G. intermedius* are the primary venomous species causing human envenomation. Although these species belong to the same genus, their envenomations induce distinct clinical symptoms, and the inconsistent efficacy of antivenoms underscores the need for species-specific therapeutic strategies. However, comprehensive molecular and toxicological profiling of Korean *Gloydius* species has remained unexplored. In this study, we quantitatively distinguished the venoms of three *Gloydius* species using two-dimensional gel electrophoresis and MALDI-TOF mass spectrometry. The results revealed that each species has evolved distinct venom protein profiles. To further investigate their expression regulation, we identified transcripts coding the major venomous proteins, which revealed species-specific regulatory differences at the transcriptional level. To verify the functionality of the identified transcripts, recombinant expression of two SVMPs was successfully expressed and their proteolytic activities were tested. This study establishes a systematic framework for understanding the molecular diversity of Korean *Gloydius* venoms and identifies species-specific toxin candidates that could serve as potential leads for the development of tailored antivenoms, diagnostic tools, and therapeutic vaccines.

**Supplementary Information:**

The online version contains supplementary material available at 10.1038/s41598-026-40454-1.

## Introduction

Snakebite incidents affect approximately 5.4 million people worldwide and result in an estimated 130,000 deaths annually, posing a serious public health concern^1^. Patients suffering from snakebite envenomation may experience cytotoxic effects, such as hemorrhage and tissue necrosis, as well as neurotoxic symptoms, including respiratory paralysis and neurological dysfunction. These clinical features can present as local or systemic symptoms and often lead to severe and long-lasting sequelae.

Snake venom is a complex mixture composed of toxic proteins, and its composition and functions have been extensively investigated over the past several decades^2–4^. In Korea, four venomous snake species inhabit the region, including *Gloydius brevicaudus*, *Gloydius intermedius*, *Gloydius ussuriensis*, and *Rhabdophis tigrinus*. Among these, the three species belonging to the genus *Gloydius* are responsible for the majority of snakebite incidents and are estimated to cause more than two thousand cases of envenomation annually^5^.

Despite their medical importance, detailed proteomic characterization of *Gloydius* snake venoms and the development of corresponding therapeutic agents remain insufficient. Recent studies have reported adverse reactions in some patients following the administration of Kovax^®^ antivenom for envenomation by *Gloydius* species^6,7^. These adverse effects include itching, urticaria, fever, vomiting, and abdominal pain, with more severe cases presenting hypotension and angioedema. Therefore, the development of more targeted antivenoms that minimize adverse reactions while improving therapeutic efficacy is urgently needed.

Snakes of the genus *Gloydius* belong to the family Viperidae and are classified within the subfamily Crotalinae pit vipers, characterized by strong hemorrhagic venom^8,9^. Their venoms predominantly contain major toxin protein families, including snake venom metalloprotease (SVMP), snake venom serine protease (SVSP), and phospholipase A_2_ (PLA_2_). Additional venom components, such as L-amino acid oxidase (M-LAO), C-type lectin (CTL), and cysteine-rich secretory protein (CRISP), have also been reported^2,10,11^. This complex mixture of toxic proteins underlies the broad spectrum of envenomation symptoms caused by *Gloydius* species, including local hemorrhage, hemolysis, and neurotoxic effects^12,13^.

Beyond their toxic properties, snake venoms have attracted considerable interest for potential therapeutic applications due to their diverse biological activities^14–17^. For example, the antihypertensive drug captopril (Capoten) was developed from a bradykinin-potentiating peptide (BPP) identified in the venom of *Bothrops jararaca*^18^. Similarly, a serine protease isolated from *Bothrops moojeni* led to the development of batroxobin, which regulates coagulation by releasing fibrinopeptide A from fibrinogen^19^. In addition, integrins derived from snake venoms have been investigated for their potential as anticancer applications owing to their ability to inhibit tumor cell adhesion to the extracellular matrix^7,20,21^. In recent years, recombinant protein production techniques have been widely adopted to enhance the therapeutic applicability of specific venom proteins^22–24^. These approaches provide valuable tools for the structural and functional characterization of venom proteins and contribute to venom-based drug discovery and development^25–27^.

In this study, we conducted a high-throughput transcriptomic and proteomic analysis of venoms from three Korean *Gloydius* species. Major venom proteins and their corresponding transcripts were identified, and these molecules were proposed as preliminary candidates for future validation as species-informative markers. In addition, full-length coding sequences of venom proteins were obtained, and one key toxin, SVMP, was successfully cloned and expressed as a recombinant protein with high yield. Collectively, these results enhance the reliability of transcriptome-based gene annotations and provide foundational data that can support the rational improvement of antivenom strategies, as well as facilitate the future development and validation of diagnostic and therapeutic applications.

## Results

### Distinct interspecific differences revealed by proteomic analysis

**Fig. 1:**
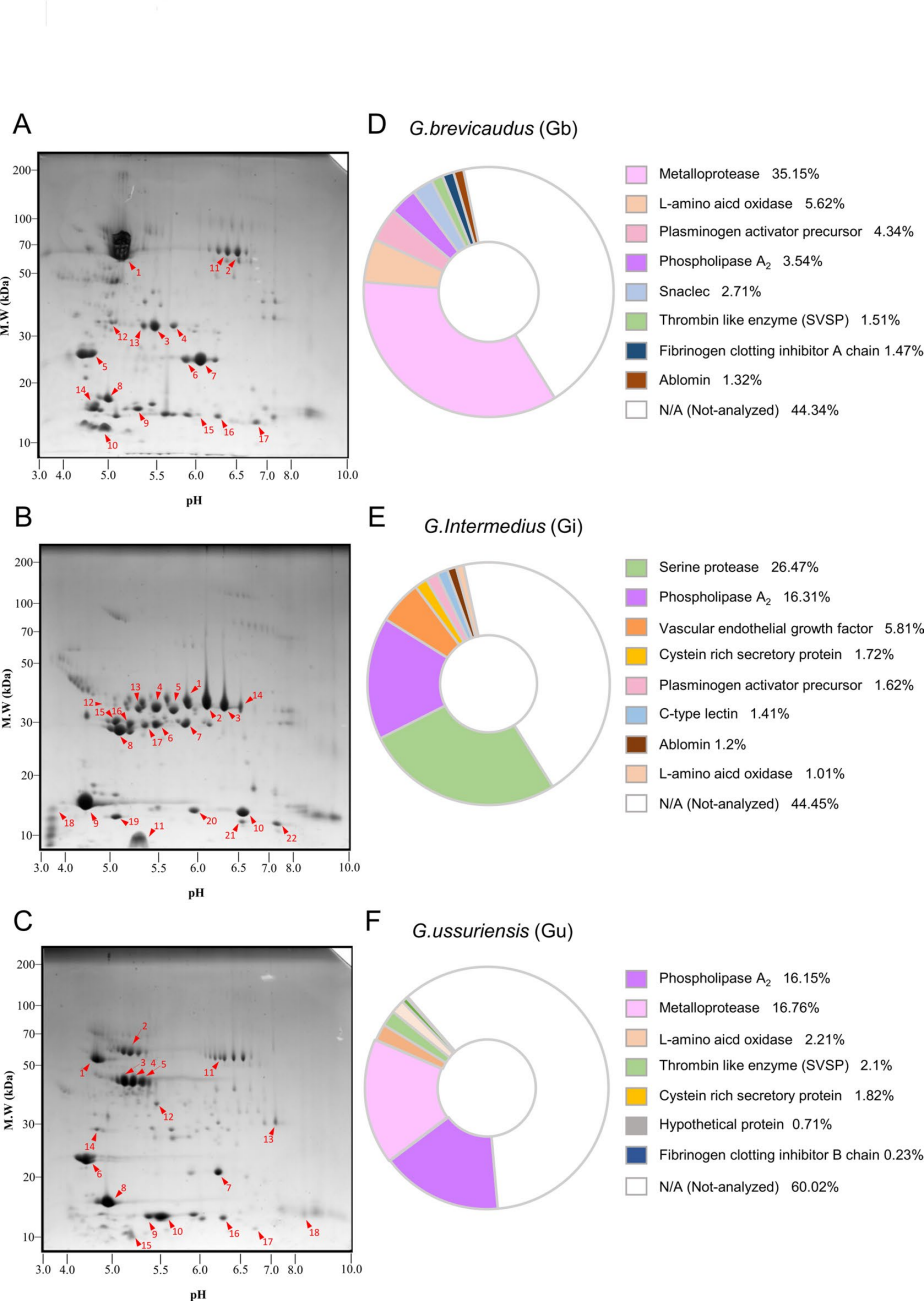
Distinct interspecific differences in venom protein composition revealed by 2-DE gel electrophoresis and quantitative analysis. Analysis of venom proteins from three Korean snake species via 2-DE. (**A**) 2-DE gel image of *G. brevicaudus*, (**B**) 2-DE gel image of *G. intermedius*, **(C)** 2-DE gel image of *G. ussuriensis*. The numbers on the spots correspond to protein IDs for MALDI-TOF MS analysis. The identified peptides for each spot are detailed in Table S1. (**D**,** E**,** F**) Protein spots of *G. brevicaudus*, *G. intermedius*, and *G. ussuriensis* were subjected to quantitative intensity analysis via ImageJ.

To compare the venom protein compositions of the three Korean *Gloydius* species, two-dimensional electrophoresis (2-DE) was performed using 1 mg of venom from each species. The resulting 2-DE gel images were visually compared to assess interspecific differences at the proteomic level (Fig. 1A–C). Overlay analysis of the three gel images revealed that only a limited number of spots were shared among the species, indicating distinct proteomic profiles. Spots exhibiting high expression levels and distinctive distribution patterns were selected from each species and are indicated by red arrows and numbers. Quantitative analysis of protein expression intensities showed that proteins with high abundance in *G. brevicaudus* and *G. ussuriensis* were broadly distributed across the molecular weight range of 10–70 kDa. In contrast, *G. intermedius* exhibited more concentrated expression patterns, mainly within the 30–40 kDa and 8–15 kDa ranges. Notably, *G. brevicaudus* and *G. ussuriensis* displayed relatively similar distributions in both molecular weight and isoelectric point (pI).

For more detailed comparison, the 2-DE profiles were subdivided into three molecular weight regions: high (H, 50–70 kDa), medium (M, 20–35 kDa), and low (L, < 20 kDa) (Supplementary Fig. 1). In the H region, *G. brevicaudus* showed strong expression of a single dominant spot (spot 1), whereas *G. ussuriensis* displayed multiple spots (spots 1–5) with variable pI values within a similar molecular weight range. In the M region, *G. brevicaudus* exhibited strong expression at relatively high pI values (spots 3, 4, 6, 7, and 13), while in the L region, species-associated protein expression was observed at lower pI values (spots 8 and 14). By contrast, *G. ussuriensis* showed pronounced expression around pI 5.5 in the L region (spots 9 and 10). *G. intermedius* exhibited distinct expression patterns across all three regions, particularly in the M region, where clusters of protein spots with similar molecular weights but varying pI values were observed. Collectively, these results suggest clear interspecific differences in venom protein composition among the three Korean *Gloydius* species. The detailed 2-DE spot patterns highlight species-associated differences in protein abundance and pI distribution, even within similar molecular weight ranges, suggesting that venom proteomic profiles provide species-informative signatures.

### Identification of key venom proteins in the three species

From the 2-DE gel images, 17 spots from *G. brevicaudus*, 22 spots from *G. intermedius*, and 18 spots from *G. ussuriensis* were selected and analyzed by matrix-assisted laser desorption/ionization time-of-flight mass spectrometry (MALDI-TOF) (Table S1). Based on the Mascot ion scores, up to three top-scoring candidate proteins were assigned to each spot. Across the three species, the identified venom proteins included snake venom metalloprotease (SVMP), snake venom serine protease (SVSP), phospholipase A_2_ (PLA_2_), L-amino acid oxidase (M-LAO), C-type lectin (CTL), cysteine-rich secretory protein (CRISP), and vascular endothelial growth factor (VEGF). Unique peptides corresponding to each protein are listed in the table together with their protein IDs and annotations. These identifications indicate that the three species exhibit distinct venom protein compositions.

For quantitative 2-DE analysis, protein spots identified by MALDI-TOF were quantified based on spot area and intensity. These spots accounted for 55.7% of the total protein spot volume in *G. brevicaudus*, 55.6% in *G. intermedius*, and 40.0% in *G. ussuriensis* (Fig. 1D–F, Table S2). When grouped by functional protein categories, *G. brevicaudus* exhibited the highest proportions of SVMP (35.15%), followed by M-LAO (5.62%), plasminogen activator precursor (4.34%), and PLA_2_ (3.54%). *G. intermedius* showed high proportions of SVSP (26.47%), PLA_2_ (16.31%), and VEGF (5.81%), whereas *G. ussuriensis* had high proportions of SVMP (16.76%), PLA_2_ (16.15%), and M-LAO (2.21%).

The venom proteomes of *G. brevicaudus* and *G. ussuriensis* showed overall similarity in that both species contained abundant SVMPs, however, notable differences were observed in the relative abundance of other venom components. SVSPs were the most abundant proteins in *G. intermedius* but were not detected as major components in the other two species. Although PLA₂ was present in all three species, its relative abundance was higher in *G. intermedius* (16.31%) and *G. ussuriensis* (16.15%) than in *G. brevicaudus* (3.54%), indicating quantitative interspecific differences. The plasminogen activator precursor was detected in *G. brevicaudus* (4.34%) and *G. intermedius* (1.62%) but was present only at minimal levels in *G. ussuriensis*, suggesting that it is not a major venom protein in that species. M-LAO was detected at levels exceeding 1% in all three species, however, its spot position (approximately 70 kDa, pI 6.5) and signal intensity differed among species, with *G. intermedius* showing a markedly weaker signal. This suggests that M-LAO may be more abundant and/or more readily detectable in *G. brevicaudus* and *G. ussuriensis* under the experimental conditions used. VEGF was uniquely detected in *G. intermedius*. In addition, vascular apoptosis-inducing protein 2 A, a member of the SVMP family, was detected only in *G. ussuriensis*.

Overall, the 2-DE gel profiles and quantitative spot analysis revealed that each of the three *Gloydius* species possesses characteristic venom protein compositions and expression patterns. *G. brevicaudus* is characterized by high proportions of SVMP, M-LAO, and plasminogen activator precursors, whereas *G. intermedius* is distinguished by the predominance of SVSP, PLA_2_, and VEGF, and *G. ussuriensis* by relatively high levels of PLA_2_ and SVMP. SVSPs (30–35 kDa, pI 5.5–6.3) were among the most prominent features in the *G. intermedius* 2-DE/MALDI-TOF dataset and are therefore proposed as candidate species-informative markers, pending validation across additional individuals and sample sets. Although it is difficult to define a single marker protein that clearly distinguishes *G. brevicaudus* from *G. ussuriensis*, the relative abundance patterns of SVMP and PLA₂ differed markedly between these species, with *G. brevicaudus* exhibiting an approximately tenfold higher proportion of SVMP relative to PLA₂ compared with *G. ussuriensis*. Taken together, SVMP, SVSP, and PLA₂ display interspecific differences in relative abundance in the present dataset and may serve as candidate species-informative markers.

### Transcriptome analysis of the three Korean ***Gloydius*** species enabled the identification of candidate venom-related transcripts

Transcriptome analysis of venom gland tissues was performed to identify transcripts encoding venom-related proteins in the three Korean *Gloydius* species. Using next-generation sequencing (NGS), 14.33 gigabases of sequence data were generated for *G. brevicaudus*, 12.65 gigabases for *G. intermedius*, and 11.7 gigabases for *G. ussuriensis*. The resulting short-read sequences were assembled de novo, yielding approximately 500,000 transcripts in total, from which about 160,000 coding DNA sequences (CDSs) were predicted across the three species. Benchmarking Universal Single-Copy Orthologs (BUSCO) analysis indicated a high level of completeness for all transcriptome assemblies (Table S3). To infer venom proteins expressed in the venom gland, the predicted CDSs were translated into amino acid sequences to construct a peptide library. This library was subsequently compared with peptide sequences identified by the proteomic analysis, with matched peptides indicated in bold in Table S1. Unique transcriptome-derived peptide matches were identified for 36 of 115 peptide fragments in *G. brevicaudus*, 49 of 107 fragments in *G. intermedius*, and 25 of 65 fragments in *G. ussuriensis*. These results indicate that a substantial proportion of venom proteins detected at the proteomic level were supported by corresponding transcriptomic evidence, confirming the successful identification of venom-related transcripts expressed in the venom glands.

### Transcriptome analysis of the three Korean ***Gloydius*** species revealed both common and distinct gene expression profiles

**Fig. 2:**
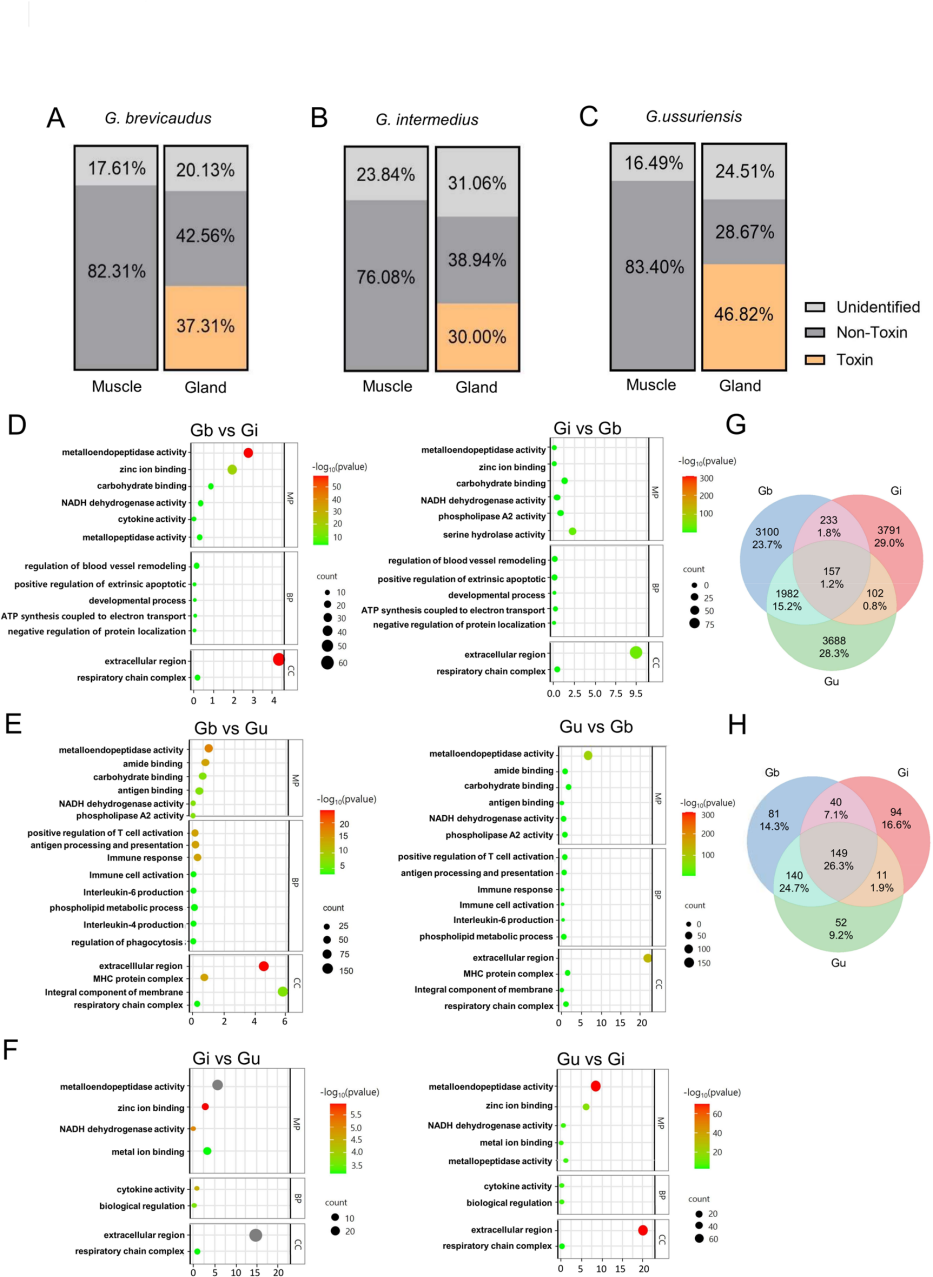
Transcriptome analysis of the three Korean *Gloydius* species revealed both common and distinct gene expression profiles. Proportional distribution of transcripts in muscle and gland tissues from (**A**) *G. brevicaudus,* (**B**) *G. intermedius,* and (**C**) *G. ussuriensis*, classified as toxin, non-toxin, or unidentified. Toxin transcript expression was notably enriched in venom gland tissues. (**D**,** E**,** F**) Gene Ontology (GO) enrichment analysis of differentially expressed transcripts in comparisons between species pairs. (**D**) *G. brevicaudus* vs. *G. intermedius*, (**E**) *G. brevicaudus* vs. *G. ussuriensis*, and (**F**) *G. intermedius* vs. *G. ussuriensis*. The bubble sizes indicate the gene count, and the color represents the adjusted *p*-value. (**G**) Venn diagram showing the number of shared and species-specific OTUs among the three species. For this analysis, OTUs that were significantly enriched in gland tissues relative to muscle (*p* < 0.05) were first selected. (**H**) Venn diagram is used to assess species specificity, with only OTUs classified as toxic protein-coding genes.

Operational taxonomic unit (OTU) genes derived from the transcriptome analysis were functionally annotated and classified into toxin and non-toxin categories according to previously established venom-toxin annotation criteria (Fig. 2A–C)^28–30^. The relative proportions of toxin-related transcripts in venom gland tissues were 37.3% in *G. brevicaudus* (Gb), 30.0% in *G. intermedius* (Gi), and 46.8% in *G. ussuriensis* (Gu) (Fig. 2A–C, Gland). In contrast, muscle tissue showed a negligible proportion of toxin-related transcripts (< 0.1%), supporting the tissue specificity of venom gene expression.

To examine interspecific differences in gene expression, transcripts showing high expression levels in venom gland tissues (*p* value < 0.05) were selected, followed by Gene Ontology (GO) enrichment analysis of differentially expressed genes (DEGs) among the three species (Fig. 2D–F). In the comparison between *G. brevicaudus* and *G. intermedius*, metalloprotease-related GO terms—including metalloendopeptidase activity (MEP), metallopeptidase activity, and zinc ion binding—were significantly enriched in *G. brevicaudus* (Fig. 2D, left column), whereas phospholipase A and serine protease–related terms were more enriched in *G. intermedius* (Fig. 2D, right column). In the comparison between *G. brevicaudus* and *G. ussuriensis*, both species showed enrichment of distinct sets of metalloprotease-related genes (Fig. 2E). Similarly, comparison between *G. intermedius* and *G. ussuriensis* revealed differences in metalloprotease-related gene enrichment, including MEP and zinc ion binding terms (Fig. 2F). However, the MEP-related DEGs identified in *G. intermedius* exhibited relatively lower statistical confidence, indicating that further investigation is required to confirm highly expressed MEP genes in this species.

In the Cellular Component category, genes associated with the extracellular region showed high enrichment and diversity across all three species, consistent with the fact that venom proteins are predominantly secreted, thereby supporting the validity of the GO enrichment analysis.

To identify species-associated transcripts, OTUs showing venom gland–specific expression relative to muscle tissue (*p* value < 0.05) were selected, and a Venn diagram was constructed to compare the three species (Fig. 2G). Transcriptome analysis identified 3,100 (23.7%), 3,791 (29.1%), and 3,688 (28.3%) gland-enriched OTUs in *G. brevicaudus*, *G. intermedius*, and *G. ussuriensis*, respectively, which were considered candidate species-associated transcripts. Approximately three-quarters of the OTUs classified as toxins were unique to individual species. By contrast, 2,139 OTUs (16.4%) were shared between *G. brevicaudus* and *G. ussuriensis*, a substantially higher number than those shared between *G. brevicaudus* and *G. intermedius* (390 OTUs) or between *G. ussuriensis* and *G. intermedius* (259 OTUs), suggesting closer transcriptomic similarity between *G. brevicaudus* and *G. ussuriensis*.

In addition, the species distribution of OTUs classified as venom protein–coding genes was examined (Fig. 2H). A total of 289 OTUs (51.0%) were shared between *G. brevicaudus* and *G. ussuriensis*, 189 OTUs (33.4%) between *G. brevicaudus* and *G. intermedius*, and 160 OTUs (28.2%) between *G. intermedius* and *G. ussuriensis*. Species-specific venom-related OTUs were identified as 81 (14.3%) in *G. brevicaudus*, 94 (16.6%) in *G. intermedius*, and 52 (9.2%) in *G. ussuriensis*. Overall, the number of shared venom-related OTUs exceeded that of species-specific OTUs, indicating a high degree of transcriptomic similarity among the three *Gloydius* species. Notably, 149 OTUs (26.3%) were commonly expressed in all three species, further supporting their close genetic relatedness.

### Functional and quantitative analysis of venom transcripts reveals the unique venom protein composition

**Fig. 3:**
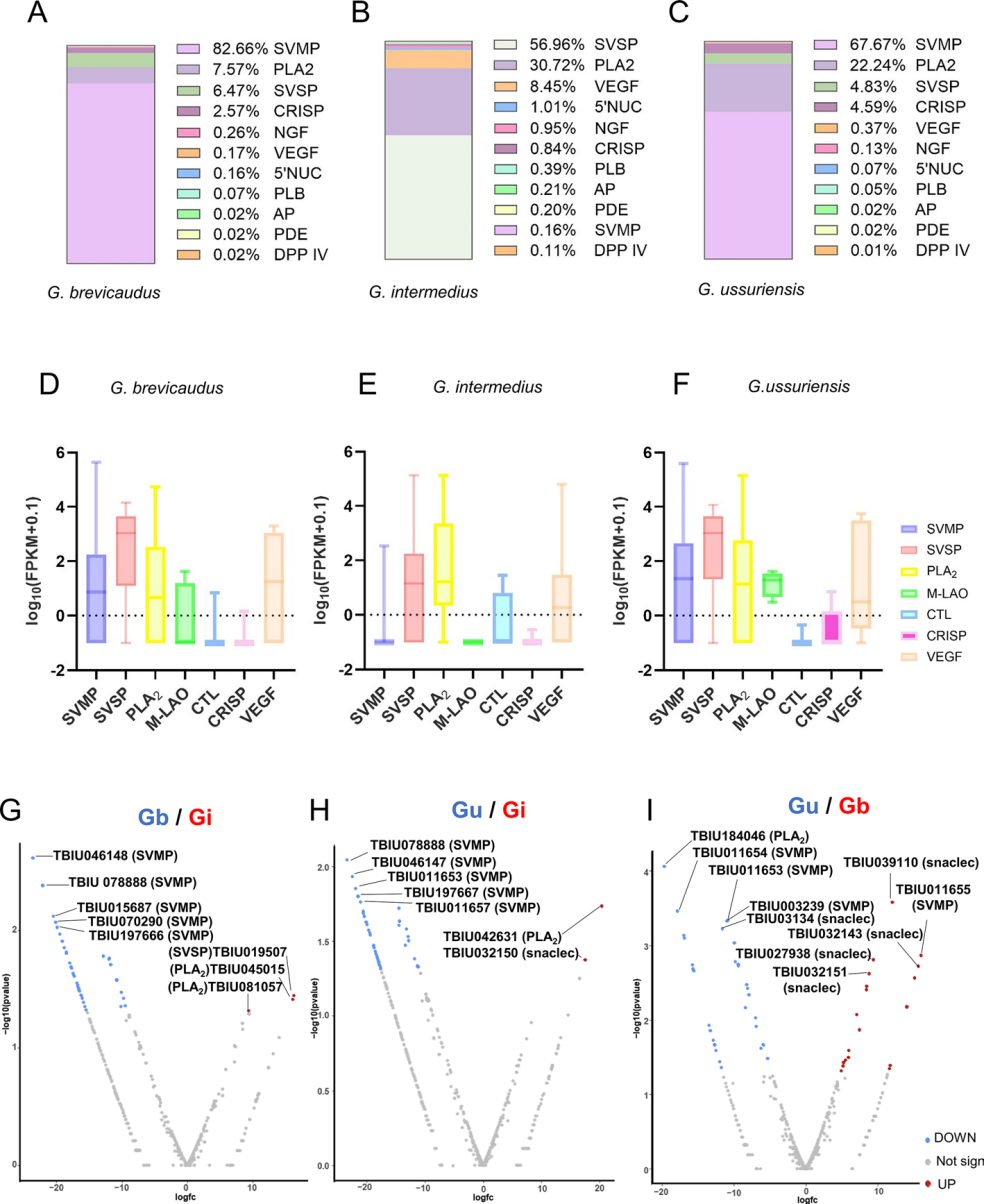
Functional and quantitative analysis of venom gene expression across three *Gloydius* species. Proportional composition of major toxin gene families in the venom gland transcriptomes of (**A**) *G. brevicaudus*, (**B**) *G. intermedius,* and (**C**) *G. ussuriensis*. (**D**,** E**,** F**) Box plots showing log(FPKM + 0.1)-transformed FPKM values of toxin gene families in each species. Each colored box represents a toxin family, highlighting interspecific variation in expression levels and distribution. Volcano plots showing differentially expressed OTUs between species pairs. (**G**) *G. brevicaudus* vs. *G. intermedius*, (**H**) *G. ussuriensis* vs. *G. intermedius* and (**I**) *G. ussuriensis* vs. *G. brevicaudus*. Significantly upregulated toxin gene candidates (adjusted *p* value < 0.05) are highlighted, demonstrating species-specific expression patterns and potential biomarker candidates. A detailed list of these OTUs is provided in Table S5.

To assess the specificity of venom-related transcripts expressed in venom glands, the transcripts were classified by protein family, and their relative abundance was calculated as a percentage of total venom transcripts for each species (Fig. 3A–C). In *G. brevicaudus*, SVMP transcripts accounted for 82.7%, followed by PLA₂ (7.6%), SVSP (6.5%), and CRISP (2.6%). In *G. intermedius*, SVSP was predominant (57.0%), followed by PLA₂ (30.7%), VEGF (8.5%), 5′-nucleotidase (5’-NUC, 1.0%), Nerve Growth Factor (NGF, 0.95%), and CRISP (0.84%). In *G. ussuriensis*, SVMP transcripts were most abundant (67.7%), followed by PLA₂ (22.2%), SVSP (4.8%), and CRISP (4.6%). Overall, SVMP transcripts were highly abundant in *G. brevicaudus* and *G. ussuriensis*, whereas SVSP transcript dominated in *G. intermedius*, and all three species exhibited consistently high levels of PLA₂ transcripts, broadly consistent with the proteomic profiles (Fig. 1).

However, notable discrepancies were observed between transcriptomic and proteomic analyses. Although SVMP transcripts were detected in *G. intermedius*, and relatively high levels of SVSP transcripts were observed in *G. brevicaudus* and *G. ussuriensis*, these patterns were not fully reflected at the protein level. In particular, SVMP transcripts accounted for 67.7% of venom-related transcripts in *G. ussuriensis*, yet the corresponding protein abundance was lower than that of PLA₂ in the proteomic analysis (Fig. 1). Such discrepancies likely reflect regulatory processes acting beyond transcription. Venom proteins must undergo translation, proper folding, post-translational modification, and trafficking through the secretory pathway before accumulating in venom, and differences in protein stability and degradation may further contribute to divergence between mRNA abundance and detected protein levels. In addition, transcriptomic profiles can be influenced by RNA integrity and transcript stability, which may vary depending on sample handling and storage conditions, whereas proteins may be preserved or degraded through distinct mechanisms. Collectively, these observations suggest that post-transcriptional and translational regulation plays an important role in shaping venom proteomes. Venom-related transcripts encoding proteins such as NGF, 5′NUC, aminopeptidase (AP), phosphodiesterase (PDE), and dipeptidyl peptidase IV (DPP IV) were detected in all three species. However, these proteins were not identified as major components in the 2-DE proteomic analysis. This discrepancy may reflect low translational efficiency or limited accumulation of these proteins in venom. Given that the 2-DE analysis detected approximately 50% of total protein spots based on intensity, it remains possible that proteins corresponding to these transcripts could be detected using more sensitive or comprehensive proteomic approaches. In contrast, transcripts encoding proteins such as M-LAO, plasminogen activator precursor, ablomin, and fibrinogen-clotting inhibitors were present at relatively low transcript levels but were identified as major venom components in the proteomic analysis, suggesting comparatively high translational efficiency or protein stability. To further resolve discrepancies between transcriptomic and proteomic datasets, additional studies incorporating whole-genome sequencing and long-read–based transcriptome analyses will be required.

To compare the transcriptional expression levels of OTUs corresponding to the seven key venom protein families identified in Fig. 1 (SVMP, SVSP, PLA₂, M-LAO, CTL, CRISP, and VEGF), FPKM values were visualized using boxplots (Fig. 3D–F). For SVMP and M-LAO, OTUs with high transcript expression were observed in both *G. brevicaudus* and *G. ussuriensis*. In the case of SVSP, several OTUs in *G. intermedius* exhibited markedly higher transcript expression than those in the other two species. In contrast, SVSP-related transcripts are likely pseudogenes or may be subject to translational silencing. PLA₂ transcript levels were comparable across all three species, however, PLA₂ protein abundance was higher in *G. intermedius* and *G. ussuriensis* than in *G. brevicaudus*. This discrepancy suggests species-dependent regulation beyond the transcriptional level, potentially involving differences in translational efficiency or protein stability. High CTL transcript expression was observed predominantly in *G. intermedius*, whereas CRISP transcripts were most prominent in *G. ussuriensis*. These transcriptomic patterns were broadly consistent with the proteomic profiles (Fig. 1).

To identify genes exhibiting relatively species-associated expression patterns, volcano plot analyses were performed (Fig. 3G–I), and venom-related genes showing significant differential expression were summarized in Table S4. In the comparison between *G. brevicaudus* and *G. intermedius*, genes with higher expression in *G. brevicaudus* were predominantly members of the SVMP family, whereas SVSP, PLA₂, and snaclec-encoding genes showed significantly higher expression in *G. intermedius* (Fig. 3G; Table S4). In comparisons involving *G. ussuriensis* and *G. intermedius*,* G. ussuriensis* exhibited relatively higher expression of SVMP genes, while *G. intermedius* showed increased expression of PLA₂ and snaclec genes (Fig. 3H).

Although *G. brevicaudus* and *G. ussuriensis* displayed overall similar proteomic and transcriptomic expression patterns, distinct OTUs within the SVMP and snaclec gene families were differentially expressed between the two species. These differences enabled the identification of OTUs that may serve as candidate species-informative markers, even between these closely related *Gloydius* species.

### Identifying the major OTUs for encoding key venom proteins

**Fig. 4:**
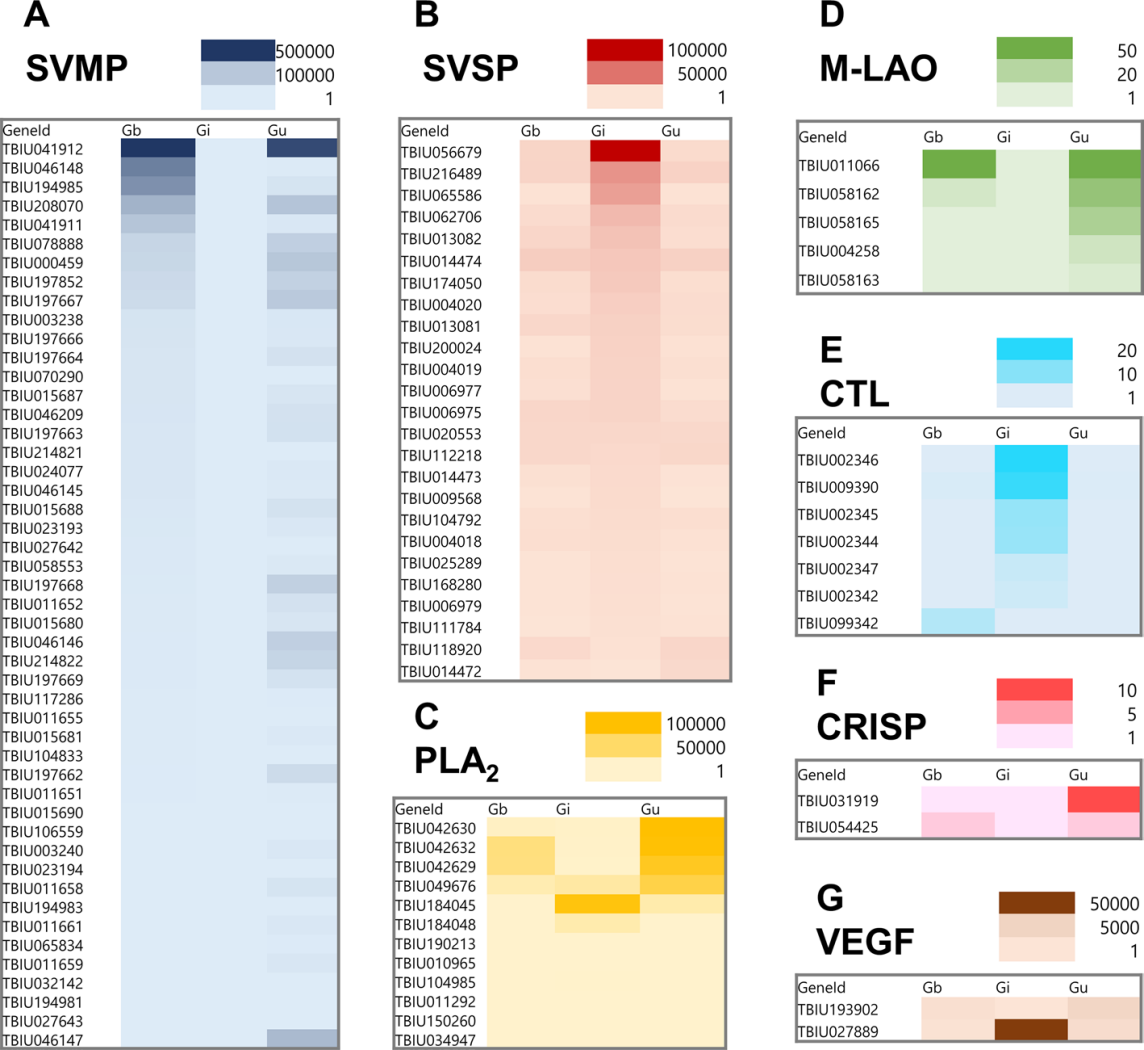
Heatmap visualization of the species-specific expression patterns of the venom gene in three *Gloydius* species. The major transcripts encoding seven venom protein families: (**A**) SVMP (**B**) SVSP (**C**) PLA_2_ (**D**) M-LAO (**E**) CRISP **(F)** CRISP and **(G)** VEGF. The expression levels of each OTU in the venom gland transcriptomes of the three *Gloydius* species are shown as FPKM values and were visualized with color gradients. Darker tiles indicate higher expression, highlighting species-specific expression patterns for several toxin candidates.

A heatmap was constructed based on the FPKM values of seven major venom gene families (SVMP, SVSP, PLA2, M-LAO, CTL, CRISP, and VEGF) expressed in the venom glands of the three *Gloydius* species (Fig. 4). SVMP OTUs were ordered based on their expression levels in *G. brevicaudus*, whereas SVSP and CTL OTUs were ordered based on their expression in *G. intermedius*. The remaining four gene families were ordered according to their expression levels in *G. ussuriensis*. *G. brevicaudus* and *G. ussuriensis* shared major venom transcript OTUs encoding SVMP, PLA₂, M-LAO, and CRISP and exhibited relatively similar expression patterns. In contrast, *G. intermedius* displayed distinct OTUs across most of the seven venom gene families. For each *Gloydius* species, OTUs showing high expression levels were identified as candidate major transcripts. Within the SVMP family, OTUs TBIU046148 and TBIU194985 were predominantly expressed in *G. brevicaudus*, whereas TBIU197668 and TBIU046146 were predominantly expressed in *G. ussuriensis* (Fig. 4A). For SVSP, OTUs TBIU056679 and TBIU065586 showed high expression specifically in *G. intermedius* (Fig. 4B). In the PLA₂ family, TBIU184045 was highly expressed in *G. intermedius*, whereas TBIU042630 showed higher expression in *G. ussuriensis* (Fig. 4C). For M-LAO, the OTU TBIU058165 exhibited elevated expression in *G. ussuriensis*. In the CTL family, OTUs TBIU002346, TBIU009390, and TBIU002345 were highly expressed in *G. intermedius*. For CRISP, TBIU031919 showed higher expression in *G. ussuriensis*, and for VEGF, TBIU027889 was predominantly expressed in *G. intermedius* (Fig. 4D–G). Collectively, these highly expressed OTUs represent candidate species-informative molecular markers, pending validation across additional individuals and sample sets.

### Recombinant expression and functional characterization of SVMPs from Korean ***Gloydius*** species via transcriptome-derived sequences

To validate transcriptomic OTUs identified by transcriptomic sequencing and to assess their functionality as venom protein–coding genes, full-length coding sequences (CDSs) were obtained and used for recombinant protein expression. Full-length sequences corresponding to highly expressed OTUs containing peptide sequences of major venom proteins were extracted from the transcriptomic data. Missing sequence regions were supplemented by referencing homologous sequences from closely related species available in the NCBI database (Table S5). Based on the assembled transcripts, gene-specific PCR primers were designed (Table S6). Full-length cDNAs were synthesized from venom gland RNA and used as templates for PCR amplification. As a result, full-length toxin gene sequences were successfully obtained for a total of 13 genes, including 3 SVMPs, 3 SVSPs, 4 PLA₂s, 2 VEGFs, and 1 M-LAO (Table 1).

**Table 1:** Full-length sequences of 15 venom toxin genes identified from venom gland transcriptomes.

Name (species)	Nucleotide sequence	Matching accession number	Identity
SVMP1 (Gb)	ATGATCCAAGTTCTCTTGGTGATTATATGCTTAGCAGCTTTTCCTTATCAAGGGAGCTCTATAATCCTGGAATCTGGGAACGTGAATGATTATGAAGTAGTGTATCCACAAAAAGTCACTGCATTGCCCAAAGGAGCAGTTCAGCCAAAGTATGAAGACGCCATGCAATATGAATTTAAAGTGAATGGAGAGCCAGTGGTCCTTCACCTGGAAAAAAATAAAGGACTTTTTTCAAAAGGTTACAGCGAGACTCATTATTCCCCTGATGGCAGAAAAATTACAACAAACCCTCCGGTTGAGGATCACTGCTATTATCATGGACGCATCCAGAATGATGCTGACTCAACTGCAAGTATCAGTGCATGCAACGGTTTGAAAGGACATTTCAAGCATCAAGGTGAGATGTACCTTATTGAACCCTTGAAGCTTTCCGACAGTGAAGCCCATGCAGTCTACAAATATGAAAACGTAGAAAAAGAGGATGAGGCCCCCAAAATGTGTGGGGTAACCCAGACTAATTGGAAATCAGATGAGCCCATCAAGGCCTCTCAGCAACAAAGATTCCCCCAAAGATACATTGAGCTTGTTGTAGTTGCAGATCATGGAATGTTCACGAAATACGACAGCAATTTAGATACTATAAGAACGTGGGTACATGAACTTGTCAACAGTATAAATGAGTTTTACAGATCTTTGAATATTGATGTCTCACTGACTGAGCTAGAAATTTGGTCCAACCAAGATTTGATCAACGTGCAGTCAGCAGCGGGTGATACTTTGGAAGCATTTGGAGACTGGAGAGAGACAGATTTGCTGAATCGCATAAGTCATGATAATGCTCAGTTACTCACGGCCACTGAATTGGATGGAAACACTATAGGATTGGCTCACGTAGCCAGCATGTGCGACCCGAAGCGTTCTACAGGAGTTGTTCAGGATCATAGTGCAATAAATCTTTTGGTTGCAGTTACAATGGCCCATGAGCTGGGTCATAATCTGGGCATGAATCATGATGGAAATCAGTGTCATTGCGGTGCTAACTCATGCGTTATGGGTGATGTACTAAGCGAAGGAGTTTCCTATGAGTTCAGTGATTGTAGTGAGAATGAATATCAGACGTATCTTACTGATCGTAACCCACAATGCATTCTCAATGAACCCTTGAGAACAGATACTGTTTCAACTCCAGTTTCTGGAAATGAACTTTTGGAGGCCGGAAAAGAATGTGACTGTGGCGCTCCTGCAAATCCGTGCTGCGATGCTGCAACCTGTAAACTGAGACCAGGGGCACAGTGTGCAGAAGGAGACTGTTGTGAGGAGTGCAGATTTATGAAAGAAGGAACAATATGCCAGGAAGCCAAGGGTGATTGGAATGATGATACCTGCACTGGCCAATCTGCTGACTGTCCCAGAAATGGCTTCTATGGCTGA	AF051790.1	98.67%
SVMP2 (Gi)	ATGATCCAAGTTCTCTTGGTGACTATATGCTTAGCAGCTTTTCCATATCAAGGGAGCTCTATAATCCTGGAATCTGGGAACGTGAATGATTATGAAGTAGTGTATCCACAAAAAGTCACTGCATTGCCCAAAGGAGCAGTTCAGCCAAAGTATGAAGACGCCATGCAATATGAATTTAAAGTGAATGGAGAGCCAGTGGTCCTTCACCTGGAAAAAAATAAAGGACTTTTTTCAAAAGATTACAGCGAGACTCATTATTCCCCTGATGGCAGAAAAATTACAACAAACCCTCCGGTTGAGGATCACTGCTATTATCATGGACGCATCCAGAATGATGCTGACTCAACTGCAAGTATCAGTGCATGCAACGGTTTGAAAGGACATTTCAAGCATCAAGGTGAGATGTACCTTATTGAACCCTTGAAGCTTTCCGACAGTGAAGCCCATGCAGTCTACAAATATGAAAACGTAGAAAAAGAGGATGAGGCCCCCAAAATGTGTGGGGTAACCCAGACTAATTGGAAATCAGATGAGCCCATCAAGGCCTCTCAGTTAGTTGTTACTCCTGAACAACAAAGATTCCCCCAAAGATACATTGAGCTTGTTGTAGTTGCAGATCATGGAATGTTCACGAAATACGACAGCAATTTAGATACTATAAGAACGTGGGTACATGAACTTGTCAACAGTATAAATGAGTTTTACAGATCTTTGAATATTGATGTCTCACTGACTGAGCTAGAAATTTGGTCCAACCAAGATTTGATCAACGTGCAGTCAGCAGCGGCTGATACTTTGGCAGCATTTGGAGACTGGAGAGAGACAGATTTGCTGAATCGCATAAGTCATGATAATGCTCAGTTACTCACGACCATTGACTTGGATGGAGACACTATAGGATTGGCTCACGTGGGCACCATGTGTGACCCAAAGTATTCTGTAGGAATTGTTCAGGATCATAGTGCAATAAACCTTTTGGTTGCAGTTACAATGGCCCATGAGCTGGGTCATAATCTGGGCATGGATCATGATGGAAATCAGTGTCATTGCGGTGCTAACTCATGCATTATGGGTGATGTACTAAGAGAAGGAGTTTCCTATGAGTTCAGTGATTGTAATAAGAATGAATATCAGACGTATCTTACTGATCGTAACCCACAATGCATTCTCAATGAACCCTTGAGAACAGATACTGTTTCAACTCCAGTTTCTGGAAATGAACTTTTGGAGGCCGGAGAAGAATGTGACTGTGGCGCTCCTGCAAATCCGTGCTGCGATGCTGCAACCTGTAAACTGAGACCAGGGGCACAGTGTGCAGAAGGAGACTGTTGTGAGCAGTGCAGATTTGTGAAAGAAGGAACAGTATGCCGGGAAGCCAAGGGTGATTGGAATGATGATTCCTGCACTGGCCAATCTGCTGACTGTCCCAGAAATGGCTTCTATGGCTGA	AF051789.1	96.47%
SVMP3 (Gu)	ATGATCCAAGTTCTCTTGGTAACTATATGCTTCGCAGTTTTTCCTTATCAAGGGAGCTCTATAATCCTGGAATCTGGGAACGTTAATGATTATGAAGTAGTGTATCCACGAAAAGTCACTGCATTGCCCAAAGGAGCAGTTCAGCCAAAGTATGAAGACGCCATGCAATATGAATTTAAAGTGAATGGAGAGCCGGTCGTCCTTCACCTGGAAAAAAATAAAGGACTTTTTTCAGAAGATTACAGCGAGACTCATTATTCCCCTGATGGCAGAGAAATTACAACATACCCCCCAGTTGAGGATCACTGCTATTATCATGGACGCATCCAGAATGATGCTGACTCAACTGCAAGCATCAGTGCATGCAATGGTTTGAAAGGACATTTCAAGCTTCAAGGGGAGATGTACCTTATTGAATCCTTGAAGCTTTCCGACAGTGAAGCCCATGCAGTCTACAAATATGAAGATGTAGAAAAAGAGGATGAGGCCCCCAAAATGTGTGGGGTAACCCAGAATTGGGAATCATATGAGCCCATCAAAAAGGCCTCTCAGTCAAATCTTACTCCTGAACAACAAACATACTTGGATGCCAAAAAATACGTTGAGTTTGTCGTAGTTCTGGACCATGGAATGTACACAAAACACAAGGACGATTTAGATAAGATAAGAACAAAAATATATGAAATTGTCAACACTATGAATGAGATGTTCATCCCTTTGAATATTCGTGTTGCACTGGTTGGCCTAGAAATTTGGTCCAACAGAGATAAGATTAACGTGACATCAGCAGCAAGTGTTACTTTGGACTCATTTAGAAACTGGAGAGCAACAGATTTGCTGAATCGCAAAATACATGATAATGCTCAGTTACTCACGACCATTGACTTGGATGGAGACACTGTAGGATTGGCTTATACGTCCGGCATGTGCCGACTGAGGCATTCTGCAGGAATTATTCAGGATCATAGCCCAATAAATCTTTTGATGGCAGTTACAATGGCCCATGAGATGGGTCATAATCTGGGCATGAATCATGACAGAGATTCCTGTACTTGTGGTGCTCCCTCATGCGTTATGGCTGACACACTAAGCCATGATCCTTCCAAACTGTTCAGCAATTGTAGTCAGGTGGATTATCGGAATAATCTTATAAAATATAGACCAAAATGCATTCTCAATGAACCCATGGGAACAGATATTGTTTCACCTCCAGTTTGTGGAAATGAACTTTTGGAGGTGGGAGAAGATTGTGACTGTGGCTCTCCTGCAAATTGTCAAAATCAGTGCTGCGATGCTGCAACGTGTAAACTGACACCAGGGTCACAGTGTGCAGATGGAGTGTGTTGTGACCAGTGCAGATTTATGGGGGCAGGAACAGAATGCCGGGCAGCAAAGGATGATTGTGACTTGCCTGAAAGGTGCACTGGCCAATCTGCTGAGTGTCCCATGGATCTCTTCCAAAGGGATGGACAACCATGCCAAAACAACTTGGGTTACTGCTACAATAGGACGTGCCCCACCATGAGGAACCAATGTATTTCTTTCCATGGGCCAAGTGCAACTGTGTCTGAAGATGCATGTTTTCAGTTTAATCTTCCGGGCAATGATCATGCCTACTGCAGAAAGGAACAAAATACAAAAATTGCATGTGAACCACAAGATGTAAAATGTGGCAGGTTATACTGCTACCGTAATTTACCCGGAAAGAGGAATATTTGCAGTGCAATATATATGCCCATGAATGAAGATATTGGGATGGTTCTTCCTGGAACAAAATGTGCAGATGGAAAGGTCTGCAGCAACGGGAAGTGTGTTGATGTGACTACAGCCTCCTGA	GQ451435.1	90.78%
PLA1 (Gu)	ATGAGGACTCTCTGGATAATGGCCGTGTTGCTGCTGGGCGTCGATGGGCACCTGCTGCAATTCAGGAAGATGATCAAGAAAATGACGGGAAAAGAGCCTGTTGTCTCCTATGCCTTTTATGGATGCTACTGCGGCTCAGGGGGCCGAGGCAAGCCAAAGGACGCCACTGACCGCTGCTGCTTTGTGCACGACTGCTGTTACGAAAAAGTGACCGGCTGCGACCCCAAATGGGACGACTACACCTACAGCTGGAAGGACGGGGATATCGTCTGTGGAGGGGACGACCCGTGCAAGAAGGAGGTTTGTGAGTGCGATAGGGCTGCGGCAATCTGCTTCCGAGACAATCTGAAGACGTACAAGAAAAGATATATGGCTTACCCGGATATTCTTTGCTCGTCAAAGTCAGAGAAATGCTGA	JX678706.1	98.37%
PLA2 (Gu)	ATGAGGACTCTCTGGATAATGGCCGTGTTGCTGCTGGGCGTCGAGGGGAGCCTGATTCAATTTGAGACCCTGATCATGAAAGTGGCGAAGAAAAGTGGTATGTTTTGGTACAACAATTATGGATGCTACTGCGGCTGGGGGGGCCAAGGCCGGCCACAGGACGCCACTGACCGCTGCTGCTTTGTGCATGACTGCTGTTACGGAAAAGTGACCGGCTGTGACCCCAAAATGGACGTCTATTCCTTCAGCGAGGAGAACGGGGATATTGTCTGCGGAGGGGACGACCCGTGCAAGAAGGAGATTTGTGAGTGCGATAGGGCCGCGGCAATCTGCTTCCGAGACAATCTGAACACATACAATGACAAAAAATATTGGGCGTTCGGGGCCAAAAATTGCCCGCAGGAGGAGTCAGAGCCATGTTGA	MW429233.1	100.00%
PLA3 (Gi)	ATGAGGACTCTCTGGATAATGGCCGTGTTGCTGCTGGGCGTCGAGGGGAGCCTGGTGCAATTCGAGACGCTGATCATGAAAATTGCGGGGAGAAGCGGTGTTTGGTACTATCGCTCTTACGGATGCTACTGTGGCGCGGGGGGCCAAGGCTGGCCACAGGACGCCAGTGACCGCTGCTGCTTTGTGCACAACTGCTGTTACAGAAAAGTGACTGGCTGCGACCCACAAATGGACGTCTACAGTTACACCGAGGAGAATGGGGATATCATCTGCGGAGGGGATGATTCGTGCCAGACACAGATTTGTGAGTGCGACAGGGCTGCGGCAATCTGCTTCCGAGATAATATGGGCACATACGACTACAAATATTGGCGGTTCTCGCCCAGAAATTGCCAGGAGGAATCAGAGCCATGCTGA	KJ654335.1	99.52%
SVSP1 (Gi)	ATGGTGCTGATCAAAGTGATAGCAAACCTTGTGATCCTACAGCTTTCTCACGCACAAAAATCTTATGAATTGATCATTGGAGGCGATGAATGTAACATAAATGAACATCGTTTCCTTGTAGCCTTGTATCACTCTAGGTCTAGGACTATGCTATGCGGTGGGACTTTGATCAACCAGGAATGGGTGCTCAGCGCTGCACACTGTGACGGGGAAGATATCCAGATAAAGCTTGGTATGCATAGCAAAAAGGTACCAAATGAGGATGAGCAGAAAAGAGTCCCAAAGGAGAAGTTCTTTTGTCTCAGTAGCAAAAACTATACCCTTTGGGACAAGGACATCATGTTGATCAGGCTGGACAGCCCTGTTAAGAACAGTACACACATCGCGCCTGTCAGCTTGCCTTCCAACCCTCCCAGTGTGGGCTCAGTTTGCCGTGTTATGGGATGGGGTACAATCACATCTCCTCAAGAGACTTATCCCGATGTCCCCCATTGTGCTAATATTAACATACTTGATTATGAGGTGTGTCAAGCAGCTCACGGAGGGTTTCCAGCAACAAGCAGAACATTGTGTGCAGGTATTCTGAAAGGAGGCAAAGATTCATGTAAGGGTGACTCTGGGGGACCCCTCATCTGTAATGGACAATTCCAGGGCATTGCATCTTGGGGGGCGCATCCTTGTGGTCAAAGTCTTAAGCCTGGTGTCTACACCAAGGTCTTCGATTATACTGAGTGGATCCAGAGCATTATTGCAGGAAATACAGATGTGTCCTGCCCCCCGTGA	KY129935.1	94.64%
SVSP2 (Gi)	ATGGTGCTGATCAAAGTGCTAGCAAACCTTCTGATACTACAGCTTTCTTACGCACAAAAATCTTCTGAACTGATCATTGGAGGTGATGAATGTAACATAAATGAACATCGTTTCCTTGTAGCCTTGTATCACTCTAGGTCTAGGACTTTGCTCTGCGGTGGGACTTTGATCAACCAGGAATGGGTGCTCAGCGCTGCACACTGTGACGGGGAAGATATCCAGATAAAGCTTGGTATGCATAGCAAAAAGGTACCAAATGAGGATGAGCAGAAAAGAGTCCCAAAGGAGAAGTTCTTTTGTCTCAGTAGCAAAAACTATACCCTTTGGGACAAGGACATCATGTTGATCAGGCTGGACAGCCCTGTTAAGAACAGTACACACATCGCACCTCTCAGCTTGCCTTCAAGCCCTCCCAGTGTGGGGTCAGTTTGCCGTATTATGGGATGGGGCAGAATCTCATCTACTAAAAAGACTTATCCCGATGTCCCTCATTGTGTTAACATTAACCTACTCGAATATGAGATGTGTCGAGTACCTTACCCAGAATTTGGGTTGCCAGCGACAAGCAGAACATTGTGTGCAGGTATCCTGGAAGGAGGCAAAGATACATGTAAGGGTGACTCTGGGGGACCCCTCATCTGTAATGGACAATTCCAGGGCATTGCATCTTGGGGAGACGATCCTTGTGCCCAACCGCATAAGCCTGCCGCGTACACCAACGTCTTCGATCATCTTGACTGGATCAAGAGCATTATTGCAGGAAATACAGATGTGTCCTGCCCCCCGTGA	KY129934.1	99.49%
SVSP3 (Gi)	ATGGTTCTGATCAGAGTGCTAGCAAACCTTCTGATACTACAGCTTTCTTACGCACAAAAGTCTTCTGAACTGATCATTGGAGGTGATGAATGTAACATAAATGAACATCGTTCCCTTGCAGTCGTGTATATCACTAGCGGTTTTCTCTGCGGTGGGACTTTGATCAACCAGGAATGGGTGCTCACTGCTGCACACTGCGACAGGGGAAATATACACATATTCCTTGGTGTGCATAGCCTAAAGGGACTAAGTAAGGATAAGCCGACAAGAATTGCAAAGGAGAAGTTCATTTGTCCCAATAGGAAAAAAGAAGACGAAAAGGACAAGGACATCATGTTGATCAGGCTGGACAGTCCTGTTAGCAATAGTGAACACATCGCACCTCTCAGCTTGCCTTCCAGCCCTCCCAGTGTGGGCTCAGTTTGCCGTGTTATGGGATGGGGCGCAATCACATCTCCTAATGAGACTTTTCCCGATGTCCCTCATTGTGCTAACATTAACATACTTGATTATGAGGTGTGTCGAGCAGCTAAACCAGAATTGCCGGCGACAAGCAGAACATTGTGTGCAGGTATCCTGGAAGGAGGCAAAGGTTCATGTTATCGTGACTCTGGGGGACCCCTCATCTGTAATGGAGAAATCCAGGGCATTGTATCTTGGGGGGGCGATATTTGTGCCCAACCGCGTGAGCCTGGCCACTACACCAAGGTCTTCGATTATATTGACTGGATCCAGAGCATTATTGCAGGAAATACAACTGTGAATTGCCCCCCGTGA	KY129936.1	98.84%
SVSP4 (Gi)	ATGGTGCTGATCAGAGTGCTAGCAAACCTTCTGATACTACAGCTTTCTTACGCACAAAAGTCTTCTGAACTGGTCGTTGGAGGTGATGAATGTAACATAAATGAACATCGTTCCCTTGTAGCCTTCTTTAACTCTACCGGGTTTTTCTGCAGTGGGACTTTGATCAACGAGGAATGGGTGCTCACCGCTGCACACTGCGACAATACAAATTTCCAGATGAAGCTTGGTGTGCATAGCAAAAAGGTACTAAATGAGGATGAGCAGACAAGAGACCCAAAGGAGAAGTTCATTTGTCCCAATAGGAAAAAAGATGACGAAAAGGACAAGGACATCATGTTGATCAGGCTGGACAGTCTTGTTAGCAACAGTGAACACATCGCGCCTCTCAGCTTGCCTTCCAGCCCTCCCAGTGTGGACTCAGTTTGCCGTATTATGGGATGGGGCACAATCAAACCTACTGAAGAGACTTATCCCGATGTCCCTCATTGTGCTAACATTAACATACTCGATCATGCGGTGTGTCGAGCAGCTTATCCAGAGTTGCTGGCGGAAAGCAGTACATTGTGTGCAGGTACCCAGCAAGGAGGCAAAGATACATGTGTGGGTGACTCTGGGGGACCCCTCATCTGTAATGGACAATTCCAGGGCATTGTATCTTATGGGGCGCATCCTTGTGGCCAAGGTCTTAAGCCTGGTGTCTACACCAAGGTCTTTGATTATAATCACTGGATTCAGAGCATTATTGCAGGAAATACAGCTGCAACTTGCCCCCCGTGA	KY129941.1	95.61%
M-LAO1 (Gb)	ATGAATGTCTTCTTTATGTTCTCACTGCTGTTCTTGGCTGCCTTGGGAAGCTGTGCAAATGACAGAAACCCCCTAGAGGAATGCTTCCGAGAAACTGACTATGAAGAATTTCTAGAGATCGCCAGAAATGGTCTGAAAGCGACATCAAACCCAAAACATGTTGTGGTTGTAGGTGCAGGAATGTCTGGGCTTAGTGCAGCCTATGTTCTTTCAGGGGCTGGACATCAGGTGACAGTTCTTGAAGCCAGTGAACGTGCGGGAGGACGAGTGAGGACTTATCGAAATGACAAAGAAGACTGGTATGCCAATCTCGGGCCCATGCGTTTACCTGAGAAACACAGGATTGTCCGGGAATATATCAGAAAGTTTGGTCTGCAGTTGAATGAATTTTCTCAGGAAAATGACAATGCCTGGTATTTTATCAAAAACATCAGGAAGAGAGTAGGGGAAGTCAAGAAAGACCCTGGCGTCTTGAAATATCCCGTGAAGCCTTCAGAAGAAGGCAAAAGTGCTGGACAGCTATATGAAGAGTCCCTCGGAAAGGTTGTAGAAGAATTAAAAAGGACTAACTGCAGCTACATACTAAATAAATATGACACCTACTCAACGAAGGAGTATCTACTTAAAGAAGGAAATCTGAGTCCTGGAGCTGTAGATATGATTGGAGACTTAATGAATGAAGATTCTGGCTATTATGTGTCTTTTCCTGAAAGCCTGAGACATGATGATATCTTTGCTTATGAAAAAAGATTTGATGAAATTGTTGGTGGAATGGATAAGTTGCCTACATCCATGTATCGAGCCATTGAGGAAAAGGTGCATTTGAATGCCCAAGTAATCAAGATACAGAAGAATGCCGAGAAAGTCACAGTGGTATATCAAACCCCAGCAAAGGAGATGGCATCTGTGACAGCTGATTATGTCATTGTGTGCACTACGTCAAGGGCCACCCGTCGCATCAAGTTTGAACCACCCCTTCCGCCAAAGAAAGCGCATGCTTTGCGGTCTGTCCACTACAGAAGTGGCACCAAGATCTTCCTCACTTGCACTAAGAAATTTTGGGAGGATGAAGGCATTCATGGTGGGAAGTCCACAACTGATCTTCCATCCCGATTCATCTACTACCCTAACCATAACTTTACTAGTGGAGTTGGGGTTATTATAGCCTATGGCATTGGTGATGATGCCAATTTCTTTCAAGCTCTTGATTTCAAGGACTGTGCTGATATTGTCATTAATGACCTTTCATTGATCCATCAGCTGCCTAGGGAAGAGATCCAGACCTTCTGTTATCCCTCAATGATTCAAAAATGGAGCCTGGATAAGTATGCTATGGGTGGTATAACCACCTTCACTCCCTACCAGTTTCAACATTTTAGTGAATCGCTCACTGCATCTGTAGACAGAATCTATTTTGCAGGGGAGCATACGGCCGAAGCTCATGGTTGGATTGACAGCACAATTAAGTCAGGGCTGAGAGCAGCAAGAGATGTGAATCGTGCTTCTGAGCAATGA	AY450403.1	99.87%
VEGF (Gb)	ATGGCTGCATACCTGCTGGCAGTTGCCATCCTCTTCTGCATCCAGGGCTGGCCATCAGGGACAGTGCAGGGACAAGTGATGCCCTTTATGGAAGTGTTCCAGCGCAGCGCCTGCCAGACCAGGGAGACGCTAGTGTCCATCCTCAAAGAGCATCCTGGTGAAATTGCCGACCTCTTCAAGCCCTCCTGTGCCACCGTGTTGCGATGCAGCGGCTGCTGCAGCGACGAAAGCCTCGCGTGCACCGCTGTGGGAAAGCGCTCCGTCGGTCGGGAGATCATGCGGGTGGATCCCCGCAAGGGGACTTCGAAGATAGAGGTGATGCAATTCACGGAGCACACAGACTGTGAATGCAGGCCTCGATCAAAAAACGGGGTGGACAACGGGGACCCCAAGAGGAACCCAGAGGAAGGGGAGCCGAGAGCCAAGTTCCCCTTTGTCTGA	AB829336.1	96.15%
VEGF (Gi)	ATGGCTGCATACCTGCTGGCAGTTGCCATCCTCTTCTGCATCCAGGGCTGGCCATCAGGGACAGTGCAGGGACAAGTGATGCCCTTTATGGAAGTGTTCCAGCGCAGCGCCTGCCAGACCAGGGAGACGCTAGTGTCCATCCTCAAAGAGCATCCTGGTGAAATTGCCGACCTCTTCAAGCCCTCCTGTGCCACCGTGTTGCGATGCAGCGGCTGCTGCAGCGACGAAAGCCTCGCGTGCACCGCTGTGGGAAAGCGCTCCGTCGGTCGGGAGATCATGCGGGTGGATCCCCGCAAGGGGACTTCGAAGATAGAGGTGATGCAATTCACGGAGCACACAGACTGTGAATGCAGGCCTCGATCAAAAAACGGGGTGGACAACGGGGACCCCAAGAGGAACCCAGAGGAAGGGGAGCCGAGGGCCAAGTTCCCCTTTGTCTGA	AB829336.1	95.92%

**Fig. 5:**
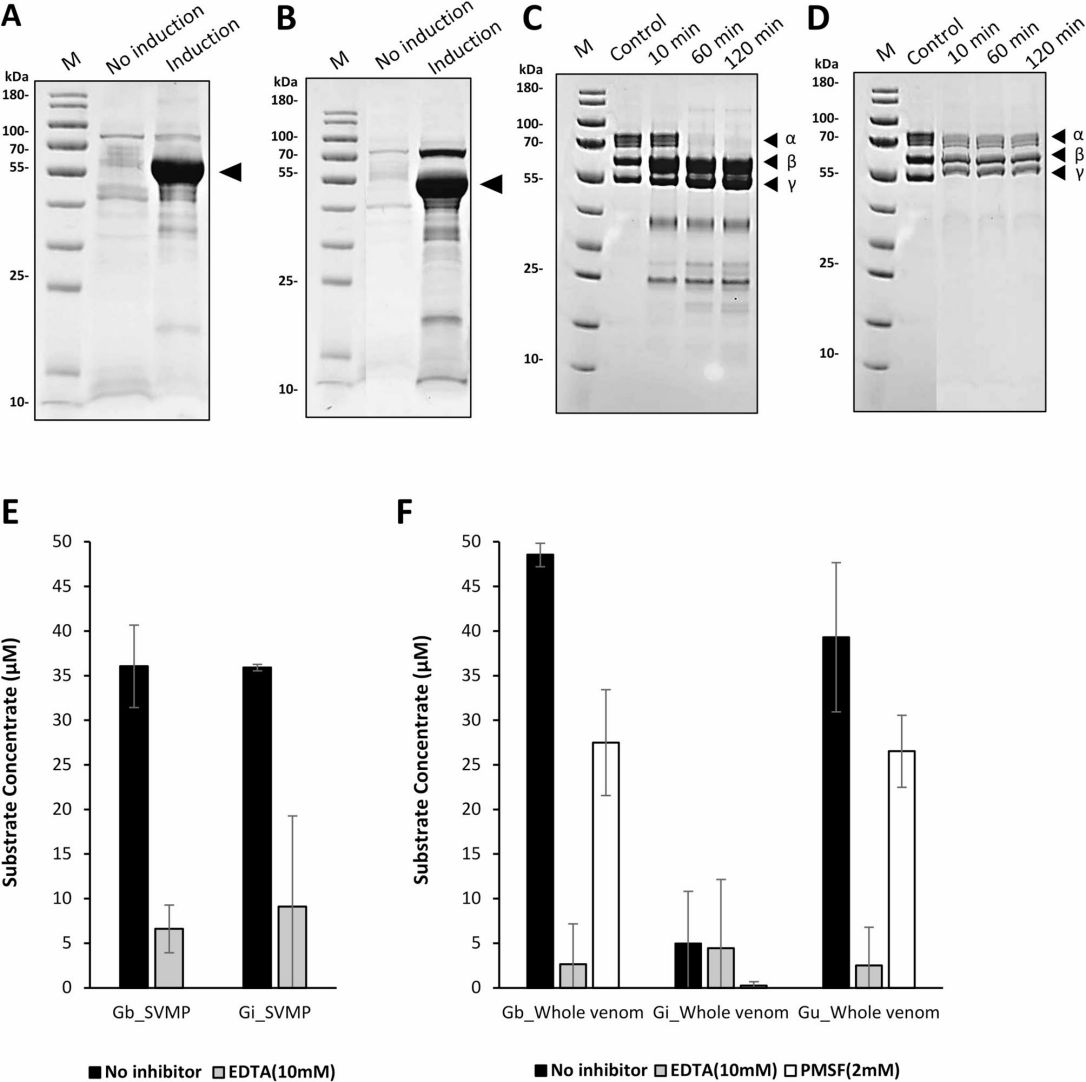
Expression and activity analysis of two recombinant SVMPs. SDS‒PAGE results showing recombinant protein expression of (**A**) Gb-SVMP and (**B**) Gi-SVMP in *Pichia pastoris*. The induced lanes show strong expression bands (indicated by arrows) corresponding to the expected molecular weights of the SVMPs. Fibrinogenolytic activity assay comparing the degradation of human fibrinogen α, β, and γ chains by (**C**) Gb-SVMP and (**D**) Gi-SVMP (0–120 min). Arrowheads indicate fibrinogen chains. (**E**) Quantitative analysis of MMP activity of recombinant SVMPs (Gb-SVMP and Gi-SVMP) at 60 min. Substrate cleavage is expressed as substrate concentration (µM). (**F**) Quantitative analysis of MMP activity of whole venoms (Gb, Gi, and Gu) at 60 min. Black, no inhibitor; light gray, EDTA (10 mM); white, PMSF (2 mM).

To functionally evaluate the identified genes, SVMP genes from two species, Gb-SVMP and Gi-SVMP, were selected for recombinant protein expression using the yeast (*Pichia pastoris*) (Fig. 5A, B). The enzymatic activities of the recombinant proteins were assessed using fibrinogenolytic assays (Fig. 5C, D) and a fluorescence-based matrix metalloproteinase (MMP) substrate assay (Fig. 5E). Recombinant Gb-SVMP exhibited strong fibrinogenolytic activity, completely degrading fibrinogen within 10 min, whereas Gi-SVMP showed no detectable fibrinogenolytic activity under the same conditions. Although fibrinogenolytic activity was observed in our assays, accurate quantification and inhibitor-based classification of the responsible proteases will necessitate additional optimization.

In an MMP substrate assay, recombinant Gb-SVMP exhibited enzymatic activity (36 µM per 3 µg over 60 min) comparable to that of recombinant Gi-SVMP (35 µM per 3 µg over 60 min) (Fig. 5E). The activities of both recombinant proteins were effectively inhibited by EDTA, confirming that substrate degradation was mediated by a metal ion–dependent enzyme family. In contrast, the proteolytic activities of whole venoms toward the MMP substrate varied markedly among species. Whole venoms from *G. brevicaudus* and *G. ussuriensis* exhibited relatively higher substrate compatibility than *G. intermedius* whole venom and were more sensitive to EDTA inhibition (Fig. 5F). These patterns are consistent with the higher abundance of SVMP family proteins in Gb and Gu venoms, supporting the comparatively lower SVMP content observed in Gi venom. When PMSF was applied to whole venoms to inhibit serine protease activity, partial inhibition of MMP substrate cleavage was observed across all three species. This finding suggests that the high MMP substrate activity of Gb and Gu whole venoms reflects cooperative contributions from both metalloproteases and other proteases, including serine proteases, rather than metalloproteases alone. Furthermore, recombinant Gb-SVMP and Gi-SVMP exhibited somewhat lower activity than an equivalent amount (3 µg) of whole venom. This difference likely reflects not only limitations associated with heterologous protein expression but also the absence of synergistic or cooperative interactions among multiple proteases that are present in native whole venoms.

## Discussion

This study integrated transcriptomic and proteomic analyses to characterize the venom protein compositions of three Korean *Gloydius* species, *G. brevicaudus*, *G. intermedius*, and *G. ussuriensis*. Two-dimensional electrophoresis combined with MALDI-TOF mass spectrometry revealed clear interspecific differences in venom protein profiles within this dataset. Seven major venom protein families, SVMP, SVSP, PLA₂, M-LAO, CTL, CRISP, and VEGF were identified as predominant components of *Gloydius* venoms. Transcriptomic analyses further supported these findings by revealing distinct gene expression patterns among the three species. In particular, *G. brevicaudus* and *G. ussuriensis* exhibited high transcript abundance of SVMPs, whereas *G. intermedius* showed predominant expression of SVSP-related transcripts. These results suggest that venom composition within the genus *Gloydius* may differ to an extent that is potentially relevant to clinical outcomes. Because the antivenom currently used in Korea is produced using venom from a single snake species, it may not fully neutralize venoms from other *Gloydius* species in some envenomation cases^6,31,32^. Adverse reactions to antivenom are commonly related to its heterologous origin and variation in patient immune responses. Together, our findings reveal proteomic differences among the three Korean *Gloydius* species and emphasize the importance of further clinical studies, such as cross-neutralization testing, to enhance antivenom performance in Korea. One limitation of this study is that our proteomic profiling relied on 2-DE followed by MALDI-TOF and Mascot database searching. This gel-based, database-dependent workflow is useful for comparing broad differences in major toxin families across species, particularly those present at high abundance. However, it does not provide de novo peptide sequencing or isoform-level resolution and may under-represent low-abundance proteins or proteins that are poorly represented in public databases. In addition, reliance on public databases with limited species-specific entries may lead to under-identification and family-level assignment for some components. Therefore, the absence or low representation of a given protein in our 2-DE and MALDI-TOF dataset should not be interpreted as a true biological absence. Additionally, some of the observed mismatches between the transcriptomic and proteomic results in this study may reflect not only biological regulation but also detection bias and identification limitations inherent to the 2-DE/MALDI-TOF–based analysis.

This study is also significant in that it employed high-throughput proteomic and transcriptomic approaches to identify potential biomarker candidates capable of distinguishing the three *Gloydius* species^14,33,34^. First, several species-associated protein spots identified on 2-DE gels exhibited distinct molecular weights, isoelectric points, or expression levels, supporting their potential value as classification markers^35–37^. Second, a subset of transcriptome-derived OTUs was matched to unique peptides identified by MALDI-TOF mass spectrometry, confirming that these transcripts represent venom proteins actively translated and accumulated in the venom gland^38–41^. In this context, species-informative peptides may serve as candidate molecular markers for comparative or classification-oriented studies, pending further validation^42,43^. Third, volcano plot and heatmap analyses enabled the selection of highly expressed and species-associated OTUs, and based on these results, SVMP was successfully expressed as a recombinant protein with experimentally verified enzymatic activity. We classified two toxins as PII based on its predicted domains. The functional validation of SVMP supports the reliability of transcriptome-derived OTU selection.

Nevertheless, interpretation of recombinant SVMP activity should be approached with caution. Heterologous expression systems may not fully recapitulate native venom gland processes, including correct protein folding, post-translational modifications such as glycosylation, and proteolytic domain maturation, all of which can influence enzymatic activity and substrate specificity. Because SVMP function is strongly affected by domain architecture and post-translational regulation, these factors should be considered when comparing recombinant protein activity with that of whole venom. Additionally, Whole venom contains multiple toxin families that can act additively or synergistically, which could in principle, make whole venom appear more active than a single recombinant component. However, in our MMP-substrate assay, Gi whole venom showed little detectable activity toward the MMP substrate. Thus, under our experimental conditions, such synergistic interactions are unlikely to be a major determinant of this specific readout. Nevertheless, synergistic interactions among venom components may still contribute to activities measured with other substrates or physiological targets, and this possibility should be considered in broader functional assessments.

At present, there are no diagnostic tools in clinical practice in Korea that can identify the snake species responsible for envenomation, and most patients visit hospitals without knowing which snake caused the bite. As a result, studies directly linking the causative snake species to clinical symptoms have been limited. Previous clinical observations have reported heterogeneous outcomes, including severe rhabdomyolysis with or without overt disseminated intravascular coagulation (DIC), despite no significant differences in time to antivenom administration^44^. Such clinical heterogeneity cannot be fully explained by treatment delay alone and suggests that interspecific differences in venom composition - such as variation in the relative abundance of toxic proteins including SVSPs - may partially contribute, although this hypothesis requires further validation. Supporting this possibility, animal studies have shown that venoms from different *Gloydius* species induce distinct neurotoxic or local tissue-damaging effects^45^. The future development of rapid species-level diagnostic assays would therefore enable integrative studies linking snake species, venom composition, and organ- or tissue-specific clinical outcomes, advancing precision diagnostics and species-informed therapeutic strategies for snakebite envenomation in Korea.

Given their high specificity and potent bioactivity, snake venom components are actively investigated as promising sources of therapeutic leads^46–48^. The findings of this study may inform future efforts to develop diagnostic assays and to prioritize targets for antivenom development or therapeutic antibody development, subject to further experimental validation and functional evaluation. An important limitation of this study is that the transcriptomic and proteomic datasets were generated from different individuals, introducing inter-individual variability and precludes strict quantitative correlations between transcript abundance and protein-level representation. Future studies incorporating paired transcriptomic and proteomic analyses from the same individuals, ideally complemented by targeted validation approaches, will be required to confirm candidate biomarkers and access their translational relevance.

Furthermore, further research should also aim to identify which venom components are directly responsible for severe clinical manifestations in envenomed patients, thereby strengthening the diagnostic and clinical significance of candidate markers. In addition to the recombinant SVMPs analyzed here, expanded functional characterization of other major venom protein families, such as SVSPs and PLA₂s, will be necessary to explore more effective therapeutic strategies for snakebite treatment^49^. In this regard, the present study provides a foundational framework for the development of venom-derived vaccines, inhibitory compounds, and diagnostic kits to improve clinical management of snakebite envenomation^50–52^.

In conclusion, this study comprehensively characterized the venom compositions of three Korean *Gloydius* species via an omics approach. It identified coding sequences of major venom proteins and proposed putative species-informative biomarker candidates. These findings offer valuable baseline data for the future development of diagnostic and therapeutic tools for snakebite management^53–55^.

## Materials and methods

### Snake tissue and venom

Venom of *Gloydius brevicaudus*, *G. intermedius*, and *G. ussuriensis* were obtained from snakes maintained at Yongmunsan Frog Snake Farm (Yangpyeong, Korea), a licensed breeding facility. A standard reference venom sample (Snake Venom, 2nd National Standard; Code No. MFDS-B-17-002) was also obtained from the Ministry of Food and Drug Safety (MFDS, Korea). The muscle and venom gland tissues for transcriptomic analysis were obtained from *Gloydius* species in Korea in September 2005 and have been stored at − 80 °C. These tissues were pre-existing materials used in this study. Therefore, no animals were sacrificed for the present study.

### Two-dimensional gel electrophoresis

Venom proteins (total 1 mg) were prepared by pooling equal amounts of venom from three individual Korean snakes (Yongmunsan Frog Snake Farm, Korea) and used for electrophoresis. Experiments were performed in two repeats (Fig. 1, Supplementary Figure S1). The protein samples were resuspended in rehydration buffer (7 M urea, 2 M thiourea, 4.5% CHAPS, 100 mM DTE, and 40 mM Tris, pH 8.8) and then applied to immobilized pH gradient (IPG) strips with a nonlinear pH 3–10 gradient (Amersham Biosciences, Uppsala, Sweden). Isoelectric focusing reached a total of 80,000 Vh. Two-dimensional SDS‒PAGE was carried out on a 9–17% linear gradient polyacrylamide gel (18 cm × 20 cm × 1.5 mm) at a constant current of 40 mA for approximately 5 h until the dye front reached the bottom of the gel. Proteins were fixed in 40% methanol and 5% phosphoric acid for 1 h and subsequently stained with Coomassie Brilliant Blue G-250 for 12 h. The gels were destained with distilled water and scanned via a Bio-Rad G710 densitometer (Richmond, CA). The scanned images were converted into digital files and analyzed via Melanie III software (GenBio, Geneva, Switzerland).

### MALDI-TOF mass spectrometry

Tryptic peptides were purified as described previously^56^. Briefly, protein spots were excised from 2-DE gels, destained with 50% acetonitrile in 25 mM ammonium bicarbonate, dried, and then rehydrated with 25 mM ammonium bicarbonate (pH 8.0) containing trypsin (50 ng) followed by incubation at 37°C for 16 h. The resulting peptides were extracted with 50% acetonitrile in 5% trifluoroacetic acid, dried, and then purified as described previously. Mass spectra were collected via a 4800 MALDI-TOF analyzer (Applied Biosystems) operated with Data Explorer software version 4.4. Spectra were generated from 1000 laser shots per spectrum within the mass range of m/z 800–4000. Mascot ion scores were calculated as − 10 × log(P), where P indicates the probability of a random peptide match. Peptides with *p* < 0.05 were considered significant and regarded as either identical or showing high sequence similarity. The identified peptides with high ion scores were used for Mascot database searches and quantification.

### Quantification of toxin groups in the proteome

Protein spots on 2-DE gels were quantified by densitometry using ImageJ software (version 1.54p). For each gel, spot intensities were measured and normalized to the total intensity of all detected spots on the same gel. Each spot was assigned to a toxin family based on MALDI-TOF peptide mass fingerprinting and Mascot identification. The relative abundance of each toxin family was then calculated as the sum of normalized intensities of all spots belonging to that family, expressed as a percentage of the total spot intensity.

### Transcriptome sequencing and analysis

Total RNA was extracted from venom glands via the standard TRIzol reagent protocol (Invitrogen)^57^. Briefly, venom gland tissues were homogenized after being finely diced, after which 20% chloroform was added to separate the RNA from the cellular debris. For further purification, RNA was isolated via RNeasy spin column purification (Qiagen). RNA samples were pooled before RNA extraction preparation for transcriptome sequencing. The concentration and purity of the extracted RNA were assessed via a NanoDrop 2000 spectrophotometer. The RNA libraries were prepared via the TruSeq Stranded mRNA Sample Prep Kit (Illumina), and sequencing was performed on an Illumina NovaSeq 6000 platform to generate 150 bp paired-end reads. The quality of the raw sequencing data was evaluated via FastQC v0.10.1^58^. Clean reads were obtained and assembled de novo via Trinity v2.0.6^59^. The completeness and accuracy of the assembly were evaluated using Benchmarking Universal Single-Copy Orthologs (BUSCO) analysis^60^. The assembled transcripts were annotated functionally via BLASTx searches (E value < 1e − 5) against the NCBI nonredundant (nr) protein database. Gene Ontology (GO) annotation was performed via Blast2GO^61^. Transcript abundance was quantified as RPKM. Venom composition at the transcript level was calculated by summing RPKM values of transcripts annotated as toxin genes within each toxin family and expressing each family as a percentage of the total RPKM of all toxin-related transcripts.

### Cloning and sequencing of venom cDNA

Based on the transcriptomic analysis results, species-specific venom protein candidate sequences were selected. To obtain the full-length coding sequences of the selected candidates, the RNAs prepared as previously described were reverse-transcribed into complementary DNA (cDNA) using the SuperScript III First-Strand Synthesis System (Invitrogen, USA). Candidate venom protein sequences were selected by integrating proteomic and transcriptomic evidence. Unique peptides identified by MALDI-TOF mass spectrometry (Table S1) were used as peptide evidence. Transcripts supported by one or more unique peptides were retained as venom candidates. To obtain full-length sequences, the corresponding complete coding sequences were retrieved using NCBI data. Then, PCR amplification was performed using the primers predicted from the transcriptomic analysis. The amplified PCR products were purified, cloned, and inserted into the pGEM-T Easy Vector System (Promega). Plasmids were extracted from individual transformed clones and subjected to Sanger sequencing by Macrogen, Inc. (Seoul, Korea)^62^.

### Recombinant protein expression in *Pichia pastoris* X-33

The SVMP gene was subsequently cloned and inserted into the pPICZαA expression vector, followed by linearization with *SacI*. The recombinant pPICZαA-SVMPs were introduced into *Pichia pastoris* X-33 by electroporation. For protein expression, the transformed cell was incubated in BMGY media (1% yeast extract, 2% peptone, 100 mM potassium phosphate buffer pH 6.0, 1.34% yeast nitrogen base, biotin, and 1% glycerol) at 30 °C, 250 rpm until the OD_600_ reached 2 to 6. Then the cells were harvested via centrifugation and resuspended in BMMY medium (containing 0.5% methanol) to an OD_600_ of 1 and incubated at 30 °C, 250 rpm for 96 h to induce protein expression. Methanol was supplemented every 24 h to a final concentration of 1%. The proteins were precipitated via the trichloroacetic acid (TCA) method and resuspended in SDS sample buffer. The expressed proteins were analyzed by SDS‒PAGE via 4–12% polyacrylamide gels and visualized via Coomassie Brilliant Blue R-250 staining.

### Fibrinogenolytic assay

To evaluate fibrinogenolytic activity, human fibrinogen (Sigma‒Aldrich, USA) was used as the substrate. The reaction mixture consisted of 50 µg of fibrinogen and 10 µg of recombinant protein in 25 mM Tris-HCl buffer (pH 6.8) in a final volume of 30 µL. The reactions were incubated at 37 °C for 10 min, 1 h, and 2 h. To terminate the reactions, SDS‒PAGE sample buffer containing β-mercaptoethanol and 1 M urea was added, followed by denaturation at 95 °C for 5 min. SDS‒PAGE was used to analyze the denatured samples, and fibrinogen degradation patterns were visualized via Coomassie Brilliant Blue R-250 staining. A negative control consisting of fibrinogen without recombinant protein was used. All experiments were performed in triplicate.

### MMP assay

Recombinant SVMPs and a generic MMP assay kit (Sensolyte, AnaSpec Inc., Fremont, CA, USA) were used. In this assay, a thiopeptolide substrate is cleaved by enzymes, liberating a sulfhydryl moiety. The assay is based on cleavage of a thiopeptolide substrate, which releases a free sulfhydryl group that reacts stoichiometrically with 5,5′-dithiobis (2-nitrobenzoic acid)(DTNB) to generate 2-nitro-5-thiobenzoic acid (TNB). TNB formation was monitored by measuring absorbance at 412 nm. The thiopeptolide substrate was diluted to a final concentration of 0.2 mM (1:50,v/v). For each well, 3 µg of recombinant SVMP (or 3 µg of whole venom for comparison) was mixed with the thiopeptolide substrate in the reaction buffer and incubated at 37°C for 60 min. To assess inhibitor sensitivity, the enzyme was preincubated for 30 min with the indicated inhibitors (10 mM EDTA, 2 mM PMSF), after which the substrate was added to initiate the reaction. After incubation, absorbance at 412 nm was measured, and the mean absorbance from replicate wells was calculated. Product formation was quantified using a standard curve generated from known concentrations, and enzymatic activity was expressed based on the calculated product concentration.

## Supplementary Information

Below is the link to the electronic supplementary material.

Supplementary Material 1

Supplementary Material 2

## Data Availability

All raw and processed sequencing data generated in this study have been submitted to the NCBI Sequence Read Archive (SRA) under accession numbers SRR35908235 (Gland tissue of *Gloydius ussuriensis*), SRR35908236 (Gland tissue of *G. intermedius*), SRR35908237 (Gland tissue of *G. brevicaudus*), SRR35908238 (Gland tissue of *G. ussuriensis*), SRR35908239 (Gland tissue of *G. intermedius*), SRR35908240 (Gland tissue of *G. brevicaudus*), SRR35908241 (Muscle tissue of *G. ussuriensis*), SRR35908242 (Muscle tissue of *G. intermedius*), SRR35908243 (Muscle tissue of *G. brevicaudus*). Published datasets analysed include BioProjects PRJNA1267255 (https://www.ncbi.nlm.nih.gov/bioproject/PRJNA1267255). Identified sequences were submitted to GenBank under accession numbers PV690027-PV690029 (Snake venom phospholipase A_2_), PV690030-PV690033 (Snake venom serineprotease), PV690034-PV690036 (Snake venom metalloprotease). Any additional information required to reanalyze the data reported in this paper is available from the lead contact upon request.
