# Supplementary material for: Comprehensive molecular characterization and comparison of venom proteins and transcripts in three Gloydius species from South Korea
Source: Sci Rep. 2026 Mar 6;16:12299. doi: 10.1038/s41598-026-40454-1 (PMC13079722; doi:10.1038/s41598-026-40454-1)
Supplement: Supplementary file 1 — Supplementary Material 1 [file 41598_2026_40454_MOESM1_ESM.docx]

**Supplementary Figure**

**
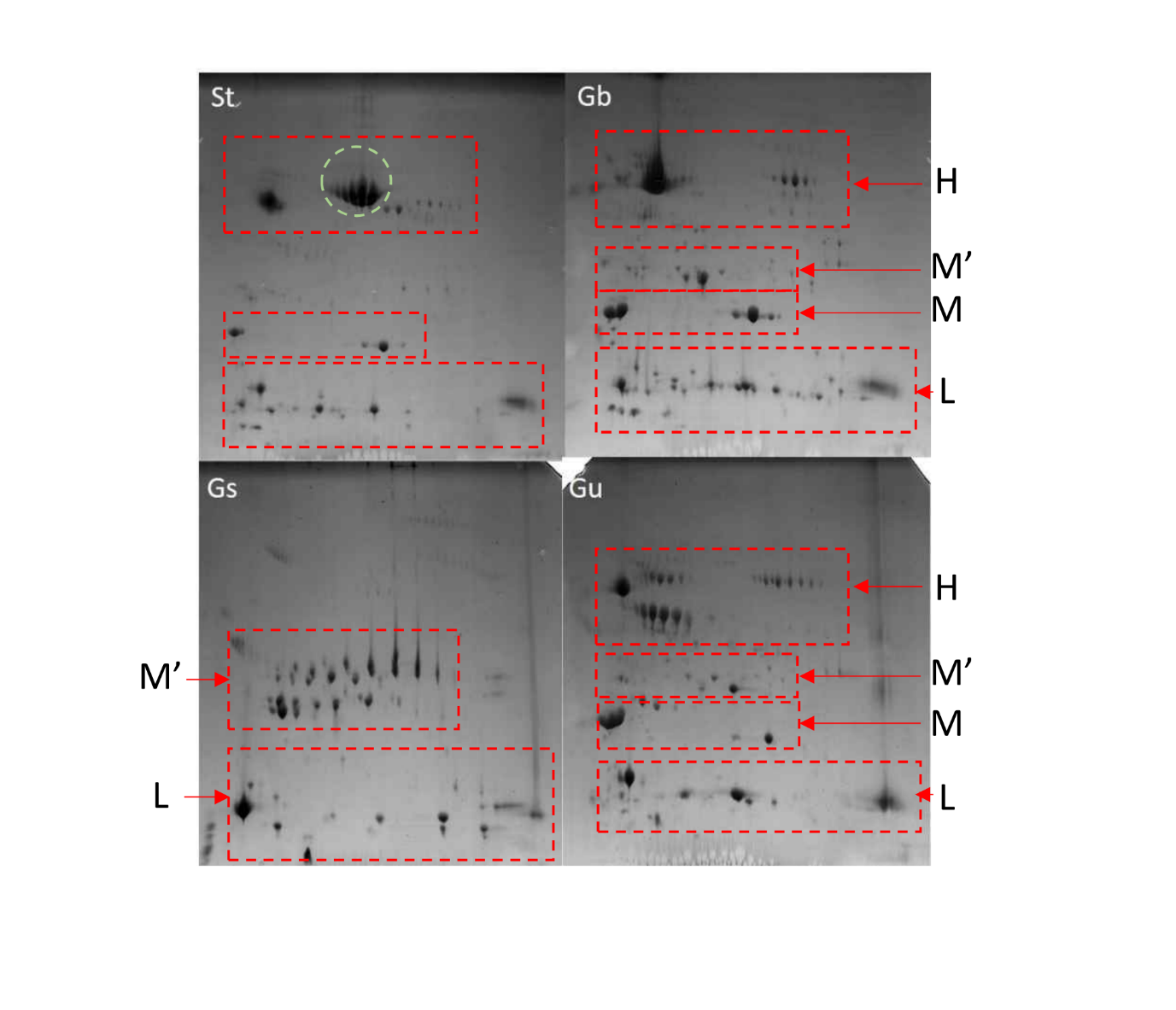
**

**Supplementary Figure1. Three Korean snake venoms (Gb, Gi, and Gu) were compared with Chinese *Gloydius* venom proteins (St)**

Chinese *Gloydius* venom proteins (St) are used for generating Kovax® antivenom. Proteins separated in 2-D PAGE were grouped in high (H), middle (M and M’), and low (L) molecular weight proteins for comparison. The green circle in the St panel represents albumin, which stabilizes venom proteins.

**Supplementary Tables**

* Supplementary tables are supplied in an Excel file with separate tabs.

**Table S1. Target proteins expected from unique peptides identified via MALDI‒TOF mass spectrometry.** Bold peptides represent unique sequences matched to transcripts identified in the assembled venom gland transcriptome (OTU). (U) indicates unique peptides found in target protein indicates unique peptides found in target protein. Matched transcript number is TBIU number.

**Table S2. Target proteins are expected from the protein identified using MALDI-TOF mass spectrometry.** Mean intensity as estimated by Image J software.

**Table S3. BUSCO analysis was used to assess the completeness and accuracy of the transcriptome assembly based on conserved single copy orthologs**

**Table S4. List of significantly differentially expressed OTUs identified in volcano plot analysis**

**Table S5. The venom coding sequence and the deduced amino acid sequence matched with the target venom protein.** Bold letters in the amino acid sequence indicate peptides found in Supplementary Table 1. Bold letters in the nucleotide sequence indicate matched sequences with mRNA sequencing.

**Table S6. Oligo list used for cloning of target venom proteins.**
